# Relevance and Recent Developments of Chitosan in Peripheral Nerve Surgery

**DOI:** 10.3389/fncel.2019.00104

**Published:** 2019-04-04

**Authors:** A. Boecker, S. C. Daeschler, U. Kneser, L. Harhaus

**Affiliations:** ^1^Department of Hand, Plastic and Reconstructive Surgery, Burn Center, BG Trauma Center Ludwigshafen, University of Heidelberg, Ludwigshafen, Germany

**Keywords:** microsurgery, peripheral nerve injuries, nerve surgery, nerve regeneration, nerve reconstruction, chitosan, nerve growth factors

## Abstract

Developments in tissue engineering yield biomaterials with different supporting strategies to promote nerve regeneration. One promising material is the naturally occurring chitin derivate chitosan. Chitosan has become increasingly important in various tissue engineering approaches for peripheral nerve reconstruction, as it has demonstrated its potential to interact with regeneration associated cells and the neural microenvironment, leading to improved axonal regeneration and less neuroma formation. Moreover, the physiological properties of its polysaccharide structure provide safe biodegradation behavior in the absence of negative side effects or toxic metabolites. Beneficial interactions with Schwann cells (SC), inducing differentiation of mesenchymal stromal cells to SC-like cells or creating supportive conditions during axonal recovery are only a small part of the effects of chitosan. As a result, an extensive body of literature addresses a variety of experimental strategies for the different types of nerve lesions. The different concepts include chitosan nanofibers, hydrogels, hollow nerve tubes, nerve conduits with an inner chitosan layer as well as hybrid architectures containing collagen or polyglycolic acid nerve conduits. Furthermore, various cell seeding concepts have been introduced in the preclinical setting. First translational concepts with hollow tubes following nerve surgery already transferred the promising experimental approach into clinical practice. However, conclusive analyses of the available data and the proposed impact on the recovery process following nerve surgery are currently lacking. This review aims to give an overview on the physiologic properties of chitosan, to evaluate its effect on peripheral nerve regeneration and discuss the future translation into clinical practice.

## Introduction

Approximately 2.8% of all hospitalized trauma patients suffer from traumatic peripheral nerve injury (Noble et al., 1998). The related severe functional impairment, as well as the consequent socio-economic impact, led to continuous research efforts in this field (Nicholson and Verma, 2004). If a direct tension free approximation of the nerve stumps is possible, to this day, the epineural nerve suture represents the first line therapy. Alternatively, if tension free coaptation is not achievable, the autologous nerve transplantation (ANT) is the current gold standard (Deumens et al., 2010). However, given the limited availability of donor nerves and the associated donor site morbidity, new approaches are needed to support in peripheral nerve surgery. Ideally, nerve conduits could provide guidance for the regenerating axons toward the distal nerve stump in the absence of negative side effects like an extended foreign body reaction or an undirected axonal regeneration. Tissue engineering utilized a considerable diversity of materials, but basic techniques like the ANT, firstly described in the 1970’s, are still up to date (Davis and Perret, 1946; Tarlov et al., 1946). Various materials have been tested for bridging peripheral nerve defects reaching from non-resorbable materials like silicon (Lundborg, 2004) to fully biodegradable materials such as Collagen or PGA (Inada et al., 2007; Bozkurt et al., 2012; Boecker et al., 2015). Today, it is a common consent that the material used to support the peripheral nerve regeneration should ideally base on a fully degradable matrix without negatively influencing the regeneration during biodegradation process (Schmidt and Leach, 2003). However, despite substantial developments in tissue engineering, there is still no material or bio-mimicking concept, which revealed superior results in peripheral nerve regeneration compared to the ANT as the current gold standard for bridging peripheral nerve defects (Deumens et al., 2010).

Besides the already established materials, Chitosan is a promising relatively novel material in the field of peripheral nerve regeneration. As it is based on the shell of arthropods it is universally available at low costs and provides a fully bioresorbable structure in the absence of toxic metabolites, potentially interfering the regeneration process (Freier et al., 2005b). Moreover, its specific physical and chemical properties enable to simulate the physiological multilayer architecture of peripheral nerves in artificial tissue engineered nerve conduits. This provides a wide field of potential applications in peripheral nerve surgery such as gap bridging, nerve suture protection or even neuroma prevention.

Previous preclinical and clinical studies investigated these conditions and demonstrated chitosan to support the axonal regeneration (Haastert-Talini et al., 2013; Stenberg et al., 2016), reduce extensive scarring, improve functional recovery (Neubrech et al., 2018) and prevent neuroma formation following peripheral nerve injury (Marcol et al., 2011). These promising preclinical and early clinical experiences in diverse nerve injury models along with its physiologic features emphasize the future potential of chitosan-based matrices in reconstructive nerve surgery. This work aims to systematically review the current tissue engineering strategies and the current process of clinical implementation.

## Properties of Chitosan and Its Impact on Peripheral Nerve Regeneration

**FIGURE 1:**
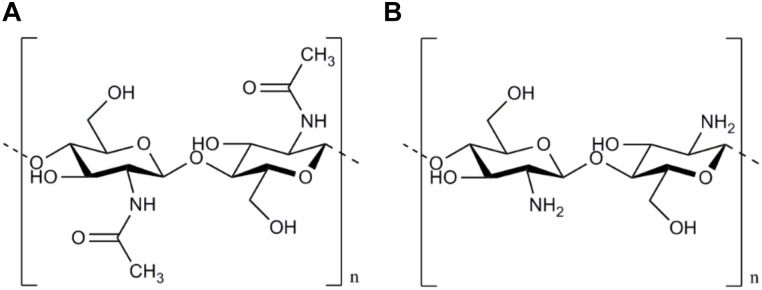
Chemical structure of Chitin and Chitosan. The chemical structure of Chitin based on a linear homopolymer of *N*-acetyl-D-glucosamine units **(A)**. After partial deacetylation chitin becomes chitosan mainly used in peripheral nerve surgery **(B)**.

The basic component of Chitosan is Chitin, a long-chain polymer of *N*-acetylglucosamine which is harvested by the exoskeletons of arthropods. After cellulose, Chitin is the second most abundant polysaccharide in nature and is aimed to be mainly used in its deacetylated modification (Chitosan) in the field of peripheral nerve surgery (Crompton et al., 2007). Chitin and chitosan can be classified into the family of the glycosaminoglycans, related to groups of chondroit sulfates, hyaluronic acid, and heparins. However, glycosaminoglycans may be the only polysaccharides with bioactive capabilities (Struszczyk, 2002). Chitin consists of a linear homopolymer of *N*-acetyl-D-glucosamine units with β-(1-4)-linkages. After partial deacetylation, chitin becomes chitosan (see Figure 1). Thus it can be can be easily obtained at very low production costs for commercial purposes by alkaline hydrolysis of chitin (Freier et al., 2005b). Depending on the processing of the chitosan, the degree of acetylation (DOA) can be varied and thus influences the molar mass as well as solvent characteristics (Chatelet et al., 2001).

Moreover, the DOA has also been shown to be a relevant factor, influencing survival, proliferation and cell activity of regeneration supporting cells like SC (Carvalho et al., 2017). But, the exact adjustments of chitosan matrices remain challenging, because the mechanical stiffness, the biodegradation time, the geometric architecture as well as the sterilization method potentially influence the axonal regeneration process and thus need to be taken into account throughout the manufacturing process (Stößel et al., 2018).

In tissue engineered nerve conduits, the biodegradation and physiologic replacement processes start immediately and thus present the central mechanisms of action which influence nerve recovery. In contrast to acid-based materials, like polyglycolic acid (PGA) or polylactide derivates, which undergo a ph decrease during biodegradation or signs of inflammatory foreign body reaction (Meek and Coert, 2008), metabolites of chitosan demonstrated neuroprotective effects during peripheral nerve regeneration.

Here, a degradation product of Chitosan, the Chitooligosaccharide (COS), has been found to promote cell proliferation and prevent apoptosis especially for SC, as the vital cell for a sufficient axonal regeneration (Zhou et al., 2008; Huang et al., 2015). Furthermore, Wang et al. (2016) attributed these stimulating effects of COS to an accelerated cell cycle leading to increased SC proliferation.

Moreover, COS stimulate the CCL2 expression by down-regulating the miR-327 of the SC, resulting in enhanced macrophage migration to the injury site (Zhao et al., 2017). In line with this, Bin et al. analyzed the anti-apoptotic effects of carboxymethylated chitosan (CMC) on SC by decreasing caspase-3, -9 and Bax activities and increasing Bcl-2 activities in CMC treated SC. To prevent the SC against oxidative stress, COS led to a reduced activity of malondialdehyde as well as to increased activity of superoxide dismutase activities (SOD) (He et al., 2014). Further *in vivo* experiments demonstrated a significantly improved peripheral nerve regeneration following daily intravenous injection COS for 6 weeks in an axonotmesis rabbit animal model. Interestingly, the number of regenerated myelinated nerve fibers, the myelin sheath thickness, as well as the compound muscle action potential (CMAP) as the parameter for electrophysiological recovery, has been shown to be significantly superior in COS treated animals. Thus, Gong et al. (2009) sum up, that COS not only accelerate the peripheral nerve regeneration but also can be seen as a neuroprotective agent after PNI.

Furthermore, the beneficial effect on neural disorders by suppression of the β-amyloid formation and supporting the anti-neuroinflammatory activity underlines the tremendous potential of COS, and consequently of chitosan, in the field of neuropathologies (Pangestuti and Kim, 2010; Hao et al., 2017).

Focusing on the tissue engineered designs, in most publications, Chitosan is the main component for a new tissue-engineered nerve conduit to bridge peripheral nerve defects. Interestingly, there are some alternative geometric structures of chitosan to promote axonal regeneration or even prevent neuroma formation.

Xu et al. (2017) have recently published another concept in the tissue engineering of chitosan by presenting a chitosan-based hydrogel in combination with a poly 3,4- ethylenedioxythiophene. Supported cell adhesion, vitality, and proliferation of PC-12 cells underline the potential of chitosan hydrogel for further neural tissue engineering. Interestingly tissue engineered concepts based on chitosan focused not only on the treatment of PNI but also for neuroma prevention. Marcol et al. (2011) applied, mircrocrystallic chitosan to the proximal nerve stump after PNI and showed less autotomy behavior and minor axonal sprouting as a key ability for neuroma prevention in the future.

## Mechanical Properties and Topographic Strategies in Tissue Engineering of Chitosan

### Mechanical Properties and Challenges

In a medical application, chitosan has already a long history due to its biocompatibility and to its non-toxic, biodegradable properties (Freier et al., 2005b).

Not only in the field of peripheral nerve surgery chitosan plays a crucial role, but also for wound healing or as a drug delivery system. Referring to the peripheral nerve, low mechanical strength has been a tremendous challenge in the early phase after implantation of chitosan and its derivates. Matrices of chitosan showed decreased stability under physiological conditions (Madihally and Matthew, 1999; Itoh et al., 2003) and therefore make it not suitable for the translational application. Modifications have been made to improve mechanical stability by designing an internal architecture by puncturing the chitosan while molding procedures with needles (Wang et al., 2006). Alternatively, Ao et al. (2006) developed a new uni-directional temperature gradient leading to a chitosan scaffolds incorporated with longitudinal microfibers. Unfortunately, the scaffold presents an appropriate mechanical strength under *in vitro* conditions but fail to show stability under physiological *in vivo* circumstances. To improve the mechanical strength of chitosan, further adjustments like additional cross-linking by supplementation of chitin (Yang et al., 2004) or formaldehyde (Desai and Park, 2005) have been published. The mechanical stability of the chitosan is also affected by the degree of acetylation (DOA). Higher DOA has been reported to improve the stability of chitosan and lead to a superior keratinocytes cell adhesion (Chatelet et al., 2001). Referring to the neural cell lineage, acetylation rates of 0.5 and 11% showed more and longer neurites of regeneration of dorsal chick root ganglions neurons compared to other concentration. However, cell viability of 0.5% was approximately eight times higher compared to a DOA of 11%. Thus, surface modifications of chitosan films referring to the cell viability and cell adhesion can clearly be modified by the DOA (Freier et al., 2005a) and needs to be addressed in the future tissue engineering for chitosan materials. In line with this, supporting cells, like SC, also benefit from a low DOA by presenting a better cell spreading and proliferation (Wenling et al., 2005).

However, for translation applications, the mechanical strength of the material to prevent the collapse of the material is essential as well as the ability for an inner lining of regenerating axons. Exemplary, Wang et al. present a chitosan two-layer approach with an oriented inner layer of nanofibers and a random outer layer with superior stability compared to random fiber mesh tubes (Wang et al., 2008a,b).

### Forms of Chitosan and Topographic Concepts

Several forms and techniques of chitosan have been published in literature reaching from hydrogels (Zheng et al., 2010; Rickett et al., 2011; Nawrotek et al., 2016b; Du et al., 2017), films (Pavinatto et al., 2010; Xiao et al., 2013; Meyer et al., 2016b), microspheres (Zeng et al., 2014) or tubes (Itoh et al., 2003; Wang et al., 2006; Shapira et al., 2016) in the field of peripheral nerve surgery.

#### Hydrogels

Hydrogels are a frequently applied form in the field of tissue engineering because of the similarity to the extracellular matrix, and the modest way of processing.

Freier et al. (2005b) presented, after the hydrolyzation of chitin, a hydrogel-based chitosan tube with superior mechanical strength, measured by the transverse compressive test. Chitosan Hydrogel has also been used as an alternative to the fibrin glue or even to the epineural suture after peripheral nerve lesion (Rickett et al., 2011). Further *in vitro* studies combine chitosan hydrogels with hydroxyapatite as a source for calcium ions to modify mechanical and biological properties. Cytotoxic and pro-inflammatory tests could mark the biocompatibility of this hydrogel solution independently of the tested composition (Nawrotek et al., 2016a).

Interestingly, *in vivo* studies with a chitosan/glycerol-beta-phosphate disodium salt hydrogel presented an impaired peripheral nerve regeneration for 10 mm sciatic nerve defect compared to chitosan tubes filled with SC suspension or culture medium (Zheng et al., 2010). Hydrogels as an inner filling in combination with chitosan tubes tested under *in vivo* conditions led to further enhanced peripheral nerve regeneration.

As drug delivery system, chitosan hydrogels have also been explored by a continuous delivery of methylprednisolone. Functional recovery of the rat’s facial (Chao et al., 2015) and sciatic nerve (Mehrshad et al., 2017) was accelerated for animals with a supplementary methylprednisolone delivery.

Hydrogels of other components, like Hyaluronic hydrogels (Meyer et al., 2016b) fibrin nanofiber hydrogels (Du et al., 2017) or simvastatin/Pluronic F-127 hydrogels (Guo et al., 2018) have been frequently combined with chitosan tubes. Axonal regeneration and motor functional recovery was improved by applying these hydrogels as inner filling for chitosan tubes.

#### Films

In the field of peripheral nerve surgery, chitosan films are applied directly for enhancing peripheral nerve regeneration but also as an inner architecture for nerve guides to bridge peripheral nerve defects. Mechanical stability and biocompatibility were proven for neural cells and peripheral nerves tissue engineering (Pavinatto et al., 2010).

Meyer et al. (2016a) combined a chitosan nerve tube with a supporting inner layer of a chitosan film over 15 mm long distance defects of a rat’s sciatic nerve lesion and presented superior axonal regeneration as well functional recovery compared to hollow non-modified chitosan tubes.

Chitosan films have been modified with several other components by crosslinking or incorporation. Proanthocyanidin was crosslinked to chitosan films with gelatine and showed improved mechanical properties, a decreased biodegradation rate, superior cell adhesion as well as improved proliferation compared to non-crosslinked gelatin or chitosan alone (Kim et al., 2005). Similar results have been found by the crosslinking of chitosan and hyaluronic in a polyelectrolyte multilayer film (Schneider et al., 2007). Incorporation of polyethylene oxid with chitosan revealed a superior water permeability and a ratio-depended antibacterial effects as well as improved mechanical strength compared to non-modified chitosan films (Zivanovic et al., 2007).

#### Microspheres

Chitosan can also be manufactured as microspheres or microparticles mainly used for drug delivery. Chitosan microspheres loaded with NGF has been incorporated to collagen-chitosan scaffolds for a rat’s sciatic nerve and led to promising results in functional outcome in combination with microchannels as inner lining (Zeng et al., 2014) as well as combined with a chitosan nerve guide conduit for reconstruction of facial nerve injuries (Liu et al., 2013). Further developments in tissue engineering enable an additional loading of cores-shell poly(lactide-co-glycolide)-chitosan microparticles as drug delivery system for continuous release of NGF (Zhang et al., 2017).

### Tubes and Inner Architecture

Different strategies of the treatment of peripheral nerve injuries based on chitosan have been explored in the literature, reaching from chitosan filament mesh tubes to chitosan-based nerve conduits. Exemplary, chitosan tubes were described by Suzuki et al. (2003) coupled with synthetized laminin under *in vivo* conditions to bridge a 15 mm sciatic nerve lesion. Despite the modification with the laminin peptides YIGSR and IKVAV, no superior results compared to the ANT have been shown (Suzuki et al., 2003). Further developments of tissue engineering led to chitosan tubes with a triangular inner surface and an additional coating with hydroxyapatite as well as laminin and laminin-1 peptides. Histological findings presented the beneficial effects of a triangular inner architecture for peripheral nerve regeneration. However, despite histological regeneration is like the ANT, delayed functional recovery was shown after 12 weeks of regeneration (Itoh et al., 2003). Addressing the essential need for inner guidance to bridge long distance peripheral nerve defects, porous chitosan tubular scaffold with chitosan fiber-based yarns with interconnected micropores were created (Wang et al., 2006). Wang et al. (2008a) also published alternative strategies for an inner lining by combining chitosan film tube as an outer layer with a nano/microfiber mesh as inner guidance. Different degrees of deacetylation were tested and compared to an ANT. Histological finding presents a better cell migration and attachment as well as neurite outgrowth for scaffolds with deacetylation rate of 93% compared to scaffolds with 78% (Wang et al., 2008a).

Furthermore, an additional mobilization with polarized β-tricalcium phosphate particles led to superior results in histological findings compared to non-polarized mesh tubes and similar results to the ANT (Wang et al., 2010). Silva et al. (2014) followed another approach presenting nanostructured hollow tubes with an additional crosslinking of genipin. Biological performance of this modified hollow tubes was assessed focussing on cell adhesion, viability, and proliferation (Silva et al., 2014). Recently, chitosan flat membranes crosslinked with dibasic sodium phosphate alone and combined with γ-glycidoxypropyltrimethoxysilan showed increased water stability and stiffness under *in vitro* conditions. For a 1 cm median nerve gap, chitosan conduits associated with γ-glycidoxypropyltrimethoxysilan promoted nerve fiber regeneration and functional recovery similar to the autograft (Fregnan et al., 2016).

Chitosan can also be combined with autologous materials by utilizing a chitosan tube with fresh skeletal muscle fibers as an inner layer to bridge nerve defects in the rat’s median nerve. Interestingly, Neuregulin-1 is upregulated for chitosan tubes with internal muscle fibers but not for hollow tubes and may be crucial to further improve axonal nerve regeneration over long nerve lesions (Ronchi et al., 2018).

### Biochemical Properties and Surface Modifications

Cell affinity for the neural cell lineage plays a crucial role to support neural regeneration. Therefore, chitosan is loaded with laminin or poly-lysine and led mainly to superior neurite outgrowth compared to the non-blended alternative (Haipeng et al., 2000). Cheng et al. (2004) applied PC12 cell culture to test cell affinity in poly-L-lysine different blended chitosan nerve tubes. Interestingly, the hydrophobicity of the poly-L-lysine in combination with the increased surface charge might be the reason for the increased cell attachment, growth, and differentiation (Cheng et al., 2004). Furthermore, increased concentration of poly-D-lysine not influence cell survival but can inhibit neurite outgrowth (Crompton et al., 2007).

Compositions of chitosan and gelatin have also been tested referring to the cell affinity and the potential to neurite outgrowth. Despite a decreased cell viability, chitosan blended with gelatine was able to show superior neurite outgrowth and in the first days of cell culture compared to PLL containing materials (Martín-López et al., 2012).

Alternative surface modification to promote peripheral nerve regeneration, are the YIGSR (Tyr-Ile-Gly-Ser-Arg) and IKVAV (Ile-Lys-Val-Ala-Val) sequences, which are known to modify receptor specific neural cell adhesion as well as to support neurite outgrowth. Itoh et al. (2005) were able to bond the YIGSR (Tyr-Ile-Gly-Ser-Arg) and IKVAV (Ile-Lys-Val-Ala-Val) peptides to molecular aligned chitosan. The peptides A3G75 and A3G83 of the laminin alpha chain LG4 modules promote neurite outgrowth. Conjugated peptides of the LG4 and LG5 modules to chitosan membranes presented improved cell attachment and neurite outgrowth for PC12 cells (Kato et al., 2002). A2G94 peptide is expressed by the Laminin alpha2 chain and led to a promoted alpha6beta1-mediated cell attachment as well as neurite outgrowth after conjugation in a chitosan tube (Urushibata et al., 2010).

Furthermore, Laminin-modified chitosan membranes significantly enhance SCs attachment and affinity for the guided peripheral nerve regeneration. Percentage of laminin incorporation is significantly higher by using the oxygen plasma technique compared to conventional chemical methods (Huang et al., 2007). Laminin has also been used to bond an additional glycin spacer to nano/microfiber mesh surface of the chitosan tube. SC affinity has been further improved by these surface modification (Huang et al., 2007). The combination of negatively charged heparin and positively charged γ-aminopropyltriethoxysilane (APTE) on porous chitosan scaffolds led to superior SC proliferation, attachment, and biological activity after seeding was shown under *in vitro* conditions (Li et al., 2015).

## Experimental Chitosan-Based Hybrid Strategies to Promote Peripheral Nerve Regeneration Under Experimental Conditions

The recent literature describes a variety of different types of hybrid strategies which aim to combine the advantages of chitosan with specific characteristics of other materials (see Table 1). The following section presents such chitosan hybrid strategies and discusses their potential role in reconstructive nerve surgery.

**Table 1:** Recently published chitosan-based hybrid models for peripheral nerve regeneration.

Nerve tube	Filler/Internal architecture/ Conduit modification	Cells or growth gactors	Nerve	Animal	Defect size (in mm)	Controls	Recovery Time in weeks	Methods	Outcome	Author
Chitosan-collagen film	–	–	Sciatic nerve	Rat	5–10	ANT	12	Electrophysiological measurements, Histological analysis	Chitosan-collagen tubes presented similar recovery for 5 mm and inferior recovery for 10 mm compared to ANT	Wei et al., 2003
Chitosan-collagen scaffold	RGD-Peptide	–	Sciatic nerve	Rat	15	ANT	8	Electrophysiological measurements, Retrograde tracing, Histological analysis, Immunochemistry	Chitosan-collagen scaffolds with RGD-Peptide modification showed superior results to non-modified scaffold but less recovery to ANT	Xiao et al., 2013
Chitosan-collagen scaffold	–	–	Sciatic nerve	beagle dog	30	ANT	12	Electrophysiological measurements, Retrograde tracing, Histological analysis, Immunochemistry	Chitosan-collagen scaffold revealed functional nerve recovery equivalent to the ANT without additional exogenous delivery or cell transplantation	Peng et al., 2018
Chitosan conduit	PGA filaments	–	Sciatic nerve	beagle dog	30	ANT	24	Electrophysiological measurements, Retrograde tracing,Histological analysis Immunochemistry	Similiar distribution patterns for myelinated axons were able to show for chitosan/PGA conduits	Wang et al., 2005
Chitosan/PGA conduit	–	–	Sciatic nerve	rat	10	ANT	12–24	Electrophysiological measurements, Retrograde tracing, Histological analysis	Even after maintained treatment after 3–6 months, chitosan/PGA conduits peripheral nerve regeneration is possible, however, an immediate repair presented superior functional results	
PLGA/chitosan nanofiber mesh tubes	Nanofibers	SC	Sciatic nerve	rat	10	PLGA/ chitosan nanofiber mesh tubes without additional cell seeding	12	Electrophysiological measurements, Histological analysis, Immunochemistry	PLGA/chitosan nanofiber mesh tubes seeded with SC led to superior results for functional recovery compared to non-seeded tubes	Zhao et al., 2014
PLGA/chitosan conduit	–	CNTF	tibial nerve	dog	25	ANT	12	Electrophysiological measurements, Histological analysis, Immunochemistry	PLGA/chitosan-CNTF presented slightly inferior recovery compared to ANT in the electrophysiological measurements and histological analysis, but better results than non-blended PLGA/chitosan	Shen et al., 2010
Chitosan- nanofiber conduit	Nanofibers with polyethylene glycol solution (PEG)	–	Sciatic nerve	rat	10	ANT	12	Histological analysis, Immunochemistry, Functional testing, Muscle Mass Measurement	Chitosan nanofiber conduit/PEG revealed superior results compared to chitosan nanofiber alone, but inferior recovery compared to ANT	Mokarizadeh et al., 2016


### Collagen

Advantages of an adjustable biodegradation time as well as the relief of non-toxic components during biodegradation, make collagen a precious and well-investigated material supporting peripheral nerve healing (Deumens et al., 2010). Considering the accepted biomimicking concept for the treatment of peripheral nerve lesions, Nawrotek et al. (2016c) developed an epineurium-mimicking chitosan conduit by a chemical composition of collagen, chitosan, and hyaluronic acid. Testing on mHippoE-18 mouse hippocampal cells, cell stimulation without cytotoxicity has been shown. As an alternative geometric design, one of the first chitosan-collagen films presented by Wei et al. (2003) showed promising results in a functional recovery for bridging peripheral nerve lesion of 5–10 mm. Progress in tissue engineering further expanded the biomimicking approach for peripheral nerve reconstruction; thus Xiao combined a hybrid nerve guide based on collagen-chitosan and added an Arg-Gly-Asp (RGD) sequence, which is a well-known pattern and mostly used for supporting cell-adhesion abilities in tissue engineering. This led to superior results in histological and functional recovery compared to nerve conduits lacking an additional RGD sequence (Xiao et al., 2013). Recently, a collagen/chitosan nerve scaffold was introduced by Peng et al. (2018) fabricated by an “unidirectional freezing process” followed by further freeze-drying (Ding et al., 2010). On the basis of a collagen and chitosan suspension, this new scaffold was applied for bridging a peripheral nerve defect of 30 mm of the beagle’s sciatic nerve. Based on the results of the electrophysiological assessment, retrograde tracing as well as histological evaluation, this collagen-chitosan nerve scaffold presented results equivalent to the ANT. If further experimental research confirms these identical recovery results via artificial conduits, such strategies will next be investigated in future clinical trials (Peng et al., 2018).

### Polyglycolic Acid (PGA)

Polyglycolic acid has been widely used in the field of bridging peripheral nerve defects. Focusing on the reconstruction of extended- distance peripheral nerve defects PGA conduits filled with laminin-soaked collagen scaffolds or PGA-collagen fibers were found to promote axonal regeneration over a nerve lesion of up to 80 mm (Toba et al., 2002). Thus, it is reasonable to combine the beneficial effects of PGA with the potential of chitosan to further optimize the regeneration supporting conduit features. Therefore, Wang et al. (2005) developed a dual-component artificial nerve graft with an outer microporous conduit of chitosan and an inner layer of filaments PGA. In a beagle dog animal model with sciatic nerve lesion of 30 mm and a recovery interval of 6 months, the chitosan/PGA group showed a similar distribution pattern of myelinated fiber diameter compared to the ANT (Wang et al., 2005).

### Polylactic Acid (PLA) and Poly(Lactic-Co-glycolic Acid) (PLGA)

The combination of chitosan and polylactide (CH-PLA) fibers presented a higher tensile strength and a lower tendency for swelling compared to chitosan fibers alone. The combination of a chitosan-based outer layer with an inner lining by (CH-PLA) fibers led to a guided axonal regeneration and thereby to the possibility to bridged long-distance peripheral nerve defects.

Furthermore, the CH-PLA fibers have been tested as a continuous release system of attached growth factors such as NGF and thereby induced a continuous outgrowth of PC12 cells as receptors for epidermal growth factor (EGF) both known to promote peripheral nerve regeneration (Huff and Guroff, 1979). Thus, CH-PLA fibers can be manufactured for gradient delivering not only for NGF but also other relevant growth factors by loading them into the alginate layers. Wu et al. emphasized the vast potential of these CH-PLA fibers, especially for a guided axonal regeneration for long-gap nerve repair (Wu et al., 2017). In line with this, Shen and colleagues bridged a peripheral nerve lesion of 25 mm of the canine tibial nerve in a dog animal model. Histological results illustrated that the PLGA/chitosan conduits were capable of providing peripheral nerve regeneration after 12 weeks. Results similar to the ANT can be reached by combining the PLGA/chitosan conduit with an additional coating of the ciliary neurotrophic factor (Shen et al., 2010). Moreover, the average maximum nerve fiber diameter and motor function have been improved by introducing dorsal root ganglion-derived SC to poly-(lactic-co-glycolic acid)/chitosan nerve scaffold for 10 mm sciatic nerve lesion in the rat (Zhao et al., 2014). However, materials based on PLA and PLGA suffer under a pH-decrease of the microenvironment during degradation and leading to an impaired of axonal regeneration (Deumens et al., 2010).

### Polyethylene Glycol

Another promising hybrid approach is to combine chitosan-based nanofiber conduits with an additional filling of polyethylene glycol solution. In a 10 mm nerve defect of the rat’s sciatic nerve, the superior functional outcome has been shown for animals treated with a substitute of the polyethylene solution. In contrast, functional recovery compared to the ANT showed clear inferior results for the experimental groups (Mokarizadeh et al., 2016). Furthermore, challenges of the chitosan in tissue engineering referring to the disadvantage in the processing of three-dimensional tubular forms without heating processing methods like extrusion or casting (Ao et al., 2006), were addressed by Nawrotek and colleagues by developing a tubular chitosan-carbon nanotube through the electrodeposition method. This conduit provided an excellent cell-adhesion, cell-proliferation, and cell-viability as well as structural stability for 28 days under *in vivo* conditions (Nawrotek et al., 2016c).

To sum up, hybrid concepts with chitosan have been well explored in literature, mostly for bridging peripheral nerve defects and led to promising results in peripheral nerve regeneration. Interestingly, the hybrid approach mainly revealed superior results of axonal recovery compared to concepts with chitosan alone. However, an exact differentiation of the effects of chitosan and the added material in a hybrid approach is not possible. Furthermore, hybrids concepts present the possibility of a tailored biodegradation as well as the embedding of growth factors or additional cell seeding related to the needs of peripheral nerve regeneration.

## Additional Cell Seeding Combined with Chitosan-Based Treatment Strategies

Nerve regeneration through chitosan-based nerve tubes can be promoted by conduit enrichment via supportive molecules like laminin or growth factors as well as additional cell seeding (see Table 2).

**Table 2:** Recently published incorporation advances of supportive cells or growth factors in the peripheral nerve system.

Nerve tube	Filler/Internal architecture/ Conduit modification	Cells or growth factor	Nerve	Animal	Defect size (in mm)	Controls	Total recovery time in weeks	Methods	Outcome	Author
Chitosan conduit	Skin fibroin filamentous fillers	Skin-derived SC	Sciatic nerve	Rat	10	Acellular nerve graft	12	Electrophysiological measurements, Retrograde tracing. Histological analysis. Immunohistochemistry, Muscle mass measurement	Histological and functional analysis were showed superior results compared to acellular nerve	Zhu et al., 2017
Laminin-modified multi -walled nerve tube	Inner layer laminin-modified chitosan; outer layer silicon	BMSC	Sciatic nerve	Rat	10	Empty silicon tube. physiological sciatic nerve	16	Histological analysis, Retrograde Tracing, Immunochemistry, Functional testing	Additional seeding with BMSC on the multi-walled nerve tube showed superior results in terms of regrowth, muscle mass of gastrocnemius, function recovery and retrograde tracing compared to empty silicon tubes	Hsu et al., 2013
Poly-3-hydroxybutyrate nerve conduit	Coating with chitosan	BMSC	Sciatic nerve	Rat	10	ANT	8	Electrophysiological measurements, Retrograde tracing, Histological analysis	Histological analysis revealed a beneficial effect of PHB/chitosan with supplementary seeding of BMSC compared to non-seeded nerve conduits. However, results still remain inferior compared to the ANT	Ozer et al., 2018
Chitosan film	Chitosan films placed around the nerve coaptation	BMSC	Sciatic nerve	Rat	–	Sciatic nerve transection and end-to-end suture	8	Electrophysiological measurements, Histological analysis, Immunochemistry, Functional testing	BMSC seeded chitosan films presented improved functional electrophysiological and histomorphometric recovery compared to non-seeded chitosan films. Results were also superior to the control group	
Chitosan/ poly(lactic glycolic acid) (PLGA)-based neural scaffold	Chitosan conduit combined with about 1000 longitudinal aligned PLGA fibers	BMSC	Sciatic nerve	Dog	50	ANT	24	Electrophysiological measurements, Retrograde tracing, Histological analysis, Muscle mass measurement	(PLGA)-based neural scaffolds seeded with BMSC indicate nerve recovery close to the ANT and better results to non-seeded nerve scaffolds, referring to the results of electrophysiological measurements and histological analysis	Ding et al., 2010
Chitosan poly(lactic-co- glycolic acid) (PLGA)-based neural scaffold	Chitosan conduit combined with about 1000 longitudinal aligned PLGA fibers	BMSC	Sciatic nerve	Dog	60	ANT	52	Retrograde Tracing, Histological analysis, Immunochemistry, Functional testing	The outcome of (PLGA)- based neural scaffolds seeded with BMSC is similar to the ANT and showed better recovery compared to non-seeded scaffold	Xue et al., 2012
Chitosan/silk fibroin nerve scaffold		Bone marrow nuclear cells	Sciatic nerve	Rat	10	ANT	12	Electrophysiological measurements. Histological analysis, Immunochemistry, Functional testing	Similar peripheral nerve regeneration of seeded chitosan/fibroin nerve scaffolds compared to the ANT and better recovery than non-seeded scaffolds	Yao et al., 2016
Autologous vein conduit combined with chitosan-β- glycerophosphate e-nerve growth factor (C/GP-NGF) hydrogel	Autologous vein graft filled with chitosan modified hydrogel	NGF	Buccal branch nerve	Rat	5	ANT	12	Electrophysiological measurements. Histological analysis. Functional testing	Autologous veins filled with (C/GP-NGF) hydrogel led to similar degree of functional and electrophysiological recovery like the ANT as well as to superior results to vein conduits blended with NGF solution	Chao et al., 2016
Chitosan conduit	NGF immobilization by Genipin cross linking	NGF	Sciatic nerve	Rat		10 ANT	24	Electrophysiological measurements. Histological analysis. Functional testing, Muscle Mass Measurements.	Considering the wet-weight ratio of the gastrocnemius muscle, the ANT presented superior results to the modified nerve conduit. Electrophysiological measurements and histological analysis revealed similar	Wang et al., 2012
GDNF- laminin blended chitosan nerve tube	Chitosan tubes blended with laminin and glial cell-line derived nerve growth	GDNF	Sciatic nerve	Rat		8 ANT	12	Functional testing. Muscle Mass Measurements	Especially sensory recovery is supported by the supplementation of GDNF to the chitosan nerve tube; Motoric recovery revealed similar in comparison to non-blended nerve tubes	Patel et al., 2007


### Additional Cell-Seeding With Schwann Cells (SC)

The proliferative effect of chitosan on SC and inhibiting effect on the fibroblast growth has already been shown by Kuang et al. (1998). Thereby, chitosan has the potential to support the axonal regeneration by increasing the number of SC and may prevent scar tissue formation after peripheral nerve lesion (Kuang et al., 1998). Further investigations regarding biocompatibility among SC chitosan scaffolds or fibers were conducted by Yuan et al. (2004), leading to the conclusion that chitosan has an excellent neuroglial cell affinity with the ability to be an excellent cell carrier system after implantation. Chitosan membranes or fibers showed almost no cell toxicity for SC considering the results in the MTT Assay (Yuan et al., 2004).

As mentioned above, the DOA of the chitosan is crucial for the solvent characteristics and molar mass. Carvalho has shown that acetylation of 5% results in higher cell proliferation and phenotypic expression of SC-like cells compared to chitosan membranes with acetylation rates of 1% or 2% (Carvalho et al., 2017). The combination of SC with a chitosan nerve conduit substituted with self-fibroin filamentous fillers showed superior regeneration results compared to non-seeded chitosan conduits regarding the histological and functional outcome (Zhu et al., 2017).

### Additional Cell-Seeding With Bone Marrow Stromal Cells (BMSC)

Bone marrow stromal cells seeded nerve conduits, in general, have shown to be a successful concept for bridging peripheral nerve defects and promoting axonal regeneration (Mimura et al., 2004; Keilhoff et al., 2006; Boecker et al., 2015). Consequently, strategies to enhance peripheral nerve regeneration by BMSC seeded chitosan-based nerve guides has also been described. Hsu et al. (2013) published a two-component nerve tube with an inner layer based on laminin-modified chitosan and a silicon-based outer layer with a supplementary seeding of BMSC. As the control group, non-seeded laminin modified-chitosan scaffolds and empty silicone tubes have been utilized. After a regeneration period of 16 weeks and following histological investigations, hyperplasia tissue enriched with eosinophils and macrophages has been found. In this context, the importance of the inflammation after PNI, induced by these cells, was emphasized by the author (Hsu et al., 2013). In line with this, a chitosan-coated poly-3-hydroxybutyrate nerve conduit has been seeded with BMSC to bridge a 10 mm sciatic nerve lesion and led to superior results in functional recovery compared to non-seeded control group. However, the ANT showed still the best results of functional recovery (Ozer et al., 2018). Similar results have been assessed through Moattari et al. (2018) by exhibiting significant superior effects in which group? in the electromyography, nerve fiber density and myelinated axon diameter (Moattari et al., 2018). For bridging long distance peripheral nerve injuries of 50 mm or even 60 mm, in a dog sciatic nerve, chitosan/poly(lactic-co-glycolic acid) (PLGA)-based neural scaffolds have been combined with BMSC and evaluated by electrophysiology, retrograde tracing, and histology after recovery of 6 or 12 months. The results led to a nearly similar degree of functional regeneration compared to the ANT (Ding et al., 2010; Xue et al., 2012). Interestingly, Yao et al. (2016) was able to show, that Bone Marrow Mononuclear Cells joined with a chitosan/silk fibroin scaffold survived at least 2 weeks under *in vivo* conditions associated with an improved axonal guidance. Nearly similar results for functional recovery was accomplished compared to the ANT in the CatWalk gait analysis after 12 weeks of recovery (Yao et al., 2016). However, SC harvested during nerve surgery need long cultivation times to reach sufficient cell numbers for reimplantation and are associated with a loss of function related to donor’s nerve.

Alternatively, BMSC can be harvested through bone marrow biopsy and revealed cell plasticity that can be adapted to the conditions of peripheral nerve regeneration (Keilhoff et al., 2006). However, the operation procedure is associated with surgical risks, like infection, bleeding, etc. Furthermore, the stability of differentiation has to be taken into account, BMSC have the ability to differentiate to SC-like cells under *in vitro* conditions but also have the capacity to re-differentiate to stem cell under the absence of the required factors, which is associated with a higher malignity degeneration rate (Gore et al., 2011).

Most current concepts of additional cell-seeding in peripheral nerve surgery remain as a proof principle because of the potential donor site morbidities, long lasting cultivation times before implantation and instability of cell differentiation (Boecker et al., 2015). Thereby, translational concepts with additional cell seeding based on chitosan are strongly restricted.

## Chitosan-Based Delivery Systems for Growth Factors

In tissue engineered delivery systems the release kinetics of growth factors play a crucial role for axonal regeneration. Recently, a variety of different concepts have been published using chitosan as a delivery system for growth factors while peripheral nerve regeneration (see Table 2).

### Nerve Growth Factor (NGF)

Pfister et al. (2008) were able to show a continuous delivery of bioactive NGF in low nanogram doses for 15 days. A nerve conduits of polyelectrolyte alginate/chitosan was coated with layers of poly(lactide-co-glycolide) (PLGA) to control the delivery of embedded NGF (Pfister et al., 2008). Moreover, the relevance of continuous delivery of NGF by using chitosan as carrying material and its synergetic effects have been marked by Chao et al. (2016). The combination of vein conduits filled with a chitosan-β-glycerophosphate-NGF hydrogel presented superior histological, functional and electrophysiological recovery compared to non-filled vein conduits (Chao et al., 2016). This underlines the capacity of chitosan to be an excellent delivering system for growth factors related to the peripheral nerve regeneration.

Furthermore, chitosan nerve conduits with a supplementary immobilization of NGF via genipin cross-linking, were compared to the ANT for a 10 mm-long sciatic nerve gap with 24 weeks recovery time. The crosslinked nerve conduit was superior compared to a non-crosslinked control but not to the ANT and led to inferior results in the electrophysiology and axon density compared to the gold standard. Thus, the real potential of additional NGF-application is hard to evaluate in this study design (Wang et al., 2012). The indication of chitosan tubes is not only limited to the treatment of PNI but can also be extended for nerve compression syndromes. Zhang et al. (2017) developed a chitosan-sericin scaffold for a continuous delivering of NGF. After application, a better functional recovery, as well as superior histological results has been demonstrated, probably caused by the beneficial effects of degradation products and the corresponding inducement of mRNA levels of GDNF, EGR2, NCAM as well as down-regulation levels of inflammatory genes (Zhang et al., 2017).

### Glial Cell Line-Derived Growth Factor (GDNF)

The beneficial effects of GDNF on the peripheral nerve regeneration, like the support of motoneuron regeneration, acceleration of axonal regeneration and the prevention of muscle atrophy are known in the literature (Chen et al., 2001; Jubran and Widenfalk, 2003). Further developments in tissue engineering led to chitosan conduits enriched with laminin and GDNF. Non-prepared laminin-loaded chitosan tubes were compared to laminin-loaded chitosan tubes with supplementation of GDNF and used to bridge a nerve lesion of 10 mm. An additional GDNF blending explicated a significant superior sensory recovery compared to unblended chitosan tubes (Patel et al., 2007).

### Fibroblast Growth Factor-2 (FGF-2)

Fibroblast growth factor-2 is one of the 22-member of the fibroblast growth factor family. In association with heparin or heparin sulfate proteoglycan, a variety of different effects has been reported including potent effects on angiogenesis and cell differentiation in the central nervous system. Chitosan-nanoparticles have been utilized as drug delivery systems for FGF-2. The chitosan scaffolds with incorporated FGF-2 microspheres reviewed superior results in cell survival and growth of neural stem cells compared to non-incorporated chitosan scaffolds under *in vitro* conditions (Woodbury and Ikezu, 2014). In line with this, chitosan scaffolds cross-linked with heparin and FGF-2 were assessed for cytocompatibility, attachment, and survival of neural stem cells (Skop et al., 2013). Alternatively, FGF-2 concentration can be increased by overpressing SC seeded in a chitosan hydrogel as an inner filling for a chitosan tube. Neural and Vascular (NVR) gel as inner filling has the obstacle to impair axonal outgrowth in a rat’s sciatic nerve model. However, this challenge could be overcome by consistent delivery of FGF-2 of overexpressing SC and led to a significant extent in neurite outgrowth compared to free FGF-2 delivery or non-modified Neonatal Rat SC (Meyer et al., 2016b).

### Platelet-Derived Growth Factor (PDGF)

Platelet-derived growth factor (PDGF) has been shown to support the migration of bone marrow stromal cells. Chitosan scaffolds have been loaded with chitosan-encapsulated PDGF microspheres and investigated for the biocompatibility of neural progenitor cells. Directional migration and growth of these cells were shown for scaffolds with additional PDGF incorporation. Chitosan microspheres underline their potential as a drug delivery system by releasing 52% of the PDGF in 4 weeks under *in vitro* conditions without a burst release (Chen et al., 2018). The beneficial effect of PDGF on neural stem progenitor cells by promoting the cell survival and cell differentiation to oligodendrocytes is described in the literature (Delayed transplantation of adult neural precursor cells promotes remyelination and functional neurological recovery after spinal cord injury; Endogenous and exogenous CNS derived stem/progenitor cell approaches for neurotrauma). In combination with chitosan channels and a continuous release of PDGF osmotic pump, neural stem progenitor cells have been significantly enhanced in cell survival under *in vivo* conditions (Guo et al., 2012).

## Translational Concepts of Chitosan-Based Nerve Tubes

Yet, the application of chitosan-based nerve conduits beneath experimental research as translational concepts has not been explored sophisticatedly. Only a few studies have already implemented chitosan tubes in clinical practice and evaluated the functional outcome after application.

In first clinical treatments, the combination of a chitosan-PGA nerve conduit was utilized to bridge a long-distance defect of 35 mm of the median nerve distal to the bicep’s aponeurosis. During a 3-year follow-up period sensory and motor function recovered satisfactorily to M4 and S3+ levels in testing. However, this case report need to be amended by further clinical studies to prove the clinical value of this nerve conduit (Fan et al., 2008). Similar results have been found by Gu et al. (2011) after bridging a 30 mm nerve defect of the median nerve with the same nerve conduit 2 years later. In June 2014, the first unaccompanied chitosan-based nerve conduit, named Reaxon^®^has been launched by Medovent GmbH (Mainz, Germany). Reaxon^®^was developed in accordance with the international standard DIN EN ISO 13485 and can be manufactured in different sizes depending on the diameter of the nerve. Nerve defects with a size up to 26 mm can be bridged in the clinical setting (Neubrech et al., 2016b). Furthermore, Reaxon^®^cannot only be used as tubulization-technique for bridging the peripheral nerve defects but also as a protector for the nerve coaptation after a tension-free epineural suture (Neubrech et al., 2016a).

**FIGURE 2:**
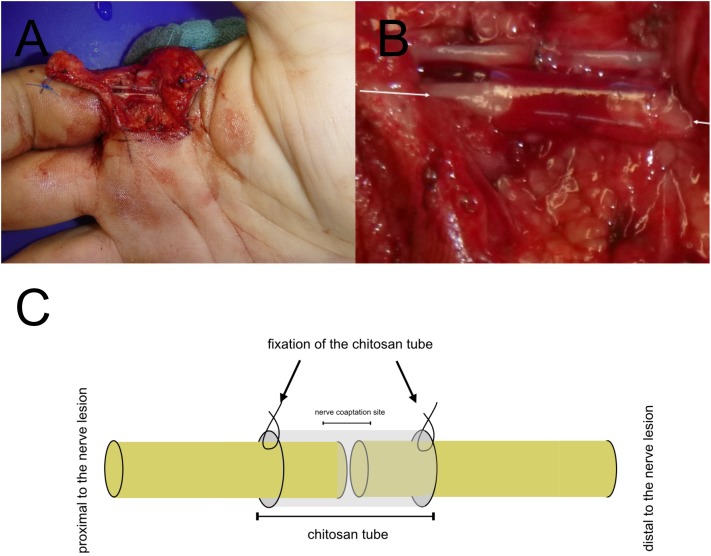
Translational Concepts in daily clinical practice. Chitosan nerve tube protects the epineural nerve coaptation **(A)**. Magnification of the chitosan-based nerve tube for covering the epineural suture of the third proper palmar digital **(B)**. Protection of the epineural nerve coaptation by a chitosan-based nerve tube in a model **(C)**. **(A,B)** has been taken during clinical routine care.

Thus, besides several studies investigated beneficial effects of chitosan and its derivates in preclinical *in vitro* and *in vivo* models, the functional outcome after traumatic sensory nerve lesions can significantly be enhanced by a supplementary use of Reaxon^®^as protection for the epineural suture in daily clinical routine (see Figure 2). In a double-blinded, randomized, prospective clinical trial, after a 6-months follow-up, a 6.3 mm two-point-discrimination has been found for patients treated with the additional chitosan-based conduit compared to the control group with two-point discrimination of 8 mm. This significant enhancement of the tactile gnosis is a relevant parameter for functional recovery of the hand (Neubrech et al., 2018). The capability of Reaxon^®^has been further investigated by Ronchi et al. by combining the chitosan tube with skeletal muscle fibers of the pectoralis muscle to bridge a 10 mm nerve lesion of the median nerve. Biomolecular analysis has shown an increased production and release of Neuregulin, as a key factor for SC survival and vitality. However, functional recovery and morphometric analyses presented no significant differences compared to hollow chitosan tubes. The author considered, beneficial effects of an inner lining by an additional muscle application is more indicated for nerve lesion of an extended distance than for a nerve lesion size of 10 mm in humans (Ronchi et al., 2018).

In the future, the possible clinical application of Chitosan in the field of peripheral nerve surgery is vast and can be used for bridging peripheral nerve lesion, protecting the epineural coaptation or even for neuroma prevention.

Referring to the findings of Marcol et al. (2011), new developments in material science led to chitosan-based nerve caps, which may have the capacity to limit neuroma formation by combining a mechanical obstacle with an additional material-related biochemical barrier for unguided axonal sprouting. Based on the material properties, recent material science concepts, such as chitosan gel, film or microcrystals, for an easier application of the material is also possible.

In conclusion, the capability of chitosan as a sophisticated partner in the peripheral nerve regeneration has been explored in several *in vitro* and *in vivo* experimental approaches. Not only the cell biocompatibility of chitosan on neural cells was shown but also beneficial effects on the differentiation and proliferation on SC as well as BMSC. Moreover, COS, as the metabolite of chitosan during biodegradation, is a neuroprotective agent during peripheral nerve regeneration and a direct stimulator for SC. Under *in vivo* conditions, chitosan was able to bridge peripheral nerve defects up to 60 mm in combination with a chitosan/poly(lactic-co-glycolic acid) (PLGA)-based neural scaffold combined with BMSC. First human trials in 2011 presented sensory recovery after chitosan tube implantation for a median nerve lesion. Recently, the first chitosan-based nerve guide was launched for clinical application and led to superior sensory recovery by using it as a biochemical protector for the site of the epineural nerve coaptation. However, given the only few human clinical trials investigating chitosan conduits, the effect on the outcome following all kinds of nerve surgery (decompression, defect reconstruction, and neuroma prevention) and thereby its potential value have to be evaluated. However, considering the first results of clinical trials and previous *in vitro* and *in vivo* experiments, chitosan has an immense potential to be a valuable partner for the peripheral nerve surgeon in the future.

## Conclusion

The relevance and application of chitosan in the field of peripheral nerve surgery are tremendous and can principally be separated between the support axonal regeneration by giving mechanical guidance and stability (as chitosan tube, inner filling or epineural suture protector) or being used as drug delivery system.

Chitosan has good biocompatibility of the peripheral nervous system, and mechanical properties make custom-made biodegradation possible. To overcome mechanical instability in a variety of different approaches in tissue engineering reaching from chitosan microspheres to chitosan hydrogel have been explored. Especially hybrid-models based on chitosan can be tailored to the demands of nerve recovery and showed promising results on all levels of peripheral nerve regeneration. In translational concepts, chitosan has been generally proven to support functional recovery, however, the clinical evidence is limited to a small number of studies yet. Future investigations should focus on the clinical outcome after application of chitosan as a drug delivery system or mechanical guidance/protection after peripheral nerve injury.

## Disclosures

Referring to Reaxon mentioned in this review, the study “Enhancing the Outcome of Traumatic Sensory Nerve Lesions of the Hand by Additional Use of a Chitosan Nerve Tube in Primary Nerve Repair: A Randomized Controlled Bicentric Trial” was sponsored by the Medovent. The study was conceived and designed before seeking financial support. Medovent was excluded from any aspect, conduct, analysis, write-up or publication of the trial. None of the authors has any personal financial related to Medovent. None of the authors has accepted compensations, fees, funding, or salary from Medovent.

## Author Contributions

AB contributed to the concept and design of the review, performed literature research, and involved in drafting and critically revising the manuscript. SD and UK involved in drafting and critically revising the manuscript. LH contributed to the concept and design of the review and involved in drafting and critically revising the manuscript. All authors read and approved the final manuscript.

## Conflict of Interest Statement

The authors declare that the research was conducted in the absence of any commercial or financial relationships that could be construed as a potential conflict of interest.

## Abbreviations

BMSC: bone marrow mesenchymal stromal cells
CCL2: chemokine ligand 2
DOA: degree of acetylation
EGR2: early growth response protein 2
FGF-2: fibroblast growth factor-2
GDNF: glial cell line-derived growth factor
NCAM: neural cell adhesion molecule
NGF: nerve growth factor
PDGF: platelet-derived growth factor (PDGF)
PGA: poly glycolic acid
PLA: polylactic acid
PLGA: poly(lactic-co-glycolic acid)
PNI: peripheral nerve injury
SC: Schwann cells
